# Object State Change Anticipation in Procedural Videos: The OSCA Task, Benchmark, and Baseline

**DOI:** 10.3390/s26144611

**Published:** 2026-07-21

**Authors:** Victoria Manousaki, Konstantinos Bacharidis, Filippos Gouidis, Konstantinos Papoutsakis, Dimitris Plexousakis, Antonis Argyros

**Affiliations:** 1 Institute of Computer Science, FORTH-Hellas, 70013 Heraklion, Greece; vmanous@ics.forth.gr (V.M.); kbach@ics.forth.gr (K.B.); gouidis@ics.forth.gr (F.G.); dp@ics.forth.gr (D.P.); argyros@ics.forth.gr (A.A.); 2 Management, Science & Technology Department, Hellenic Mediterranean University, 72100 Agios Nikolaos, Greece; 3 Computer Science Department, University of Crete, 70013 Heraklion, Greece

**Keywords:** object state change anticipation, video understanding, visual-semantic features, egocentric procedural videos

## Abstract

Anticipating how objects will change state during ongoing activities is essential for robust video understanding and for deploying assistive, AR, and robotics systems in the real world. We introduce Object State Change Anticipation (OSCA), the problem of predicting the imminent state change of an object at the onset of the next, yet unobserved, action in egocentric procedural videos. To facilitate research on this task, we present Ego4D-OSCA, a curated benchmark that augments Ego4D segments with consistent pre-/post-state labels, inverse state relations, and a “no state change” category. Building on this dataset, we propose the first baseline for OSCA that fuses recent egocentric visual context with a structured linguistic history of actions and object states, enabling temporally grounded reasoning about near-future transformations. We establish comprehensive evaluation protocols and report extensive experiments, including ablations on modality contributions, temporal context, and robustness to class imbalance and long-tail distributions. Results demonstrate that (i) integrating structured action/state history significantly improves anticipation over visual-only variants, (ii) modeling inverse state relations benefits generalization across diverse objects and contexts, and (iii) OSCA is complementary to action anticipation and next-active object prediction, targeting a distinct predictive capability. We release Ego4D-OSCA together with code, baseline models, and reproducible training scripts to facilitate fair comparison and future advances.

## 1. Introduction

When observing human–object interactions, we can effortlessly reason about and anticipate actions and their effects as changes in object states [1,2,3]. Imagine, for example, that someone brings a bottle of wine to the dinner table. Even before opening it, we can infer that in the near future, the bottle will be “opened” and glasses will be “filled”. Recognizing and anticipating object states and their changes is crucial for any entity that affects its physical and functional properties and plays a decisive role in activity understanding, reasoning, and task planning. While intuitive for humans, predicting changes in the state of objects remains beyond the capabilities of current AI systems [4,5]. Although related tasks, such as visual object perception, next-active object prediction, and action recognition, have been studied extensively, the explicit anticipation of object state changes remains largely unexplored. Yet, this capability is vital for enabling AI agents to understand and assist in procedural activities [6,7].

The problem of Object State Classification (OSC) is defined as the multi-class recognition of an object’s state in a still image [8,9], or the initial and the final object states in a video that demonstrates one or more state-modifying actions [10]. The binary object state change classification variant is also related to detecting state change occurrences in a short video clip [10,11]. Researchers have only recently begun to focus on methods for representing and understanding object state changes in videos within the context of state-modifying actions [12,13,14], which can also be viewed as transformations [15]. Existing benchmarks largely ignore object state changes and focus on traditional types of annotations related to object type, location, or shape, attributes, affordances, and human actions.

**Figure 1:**
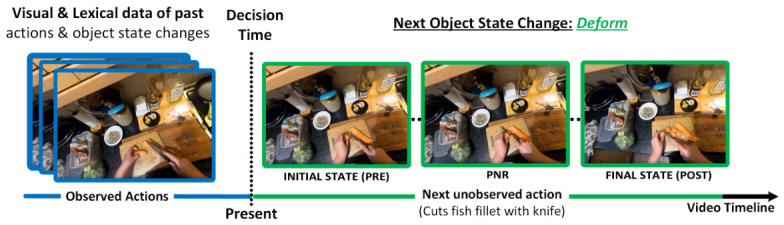
We introduce the new problem of anticipating object state changes (OSCA) in procedural videos, where the goal is to predict upcoming object state changes (if any) at the onset of the next action. An object state change refers to a physical and possibly functional change in an object’s attributes and is based on the transition from a pre-state (initial) to a post-state (final) occurring at the Point of No Return (PNR) time. Images acquired from the Ego4D database (“Egocentric Live 4D Perception (Ego4D) Database: A Ego4D Consortium 2020”, https://ego4d-data.org/ (accessed on 25 May 2026).

In this work, we take one step beyond the Object State Classification and the Action Anticipation tasks by introducing the new task of Object State Change Anticipation (OSCA) in videos. OSCA focuses on the multi-class prediction of the state change that occurs on an object during the next, yet unseen at inference time, action in a long procedural activity. Specifically, as shown in Figure 1, at a certain decision point in time that is at the start of the next, yet unobserved action, we aim to predict the object state change class that will occur. The object state change will occur at the “Point of No Return” timestamp during the next action [10].

The proposed Object State Change Anticipation task is related to, but fundamentally distinct from, existing formulations of action anticipation and next-active object prediction. While action anticipation focuses on estimating the future human action label, OSCA instead aims to forecast the imminent transformation of the physical world induced by that action, i.e., the anticipated object state transition. Formally, given an observed procedural video sequence $V_{1:t}$ together with the history of previous actions $A_{1:k}$ and object state transitions $S_{1:k}$, OSCA aims to estimate the probability distribution of the next object state transition $s_{k+1}$: $P(s_{k+1}|V_{1:t},A_{1:k},S_{1:k})$ In contrast, conventional action anticipation estimates: $P(a_{k+1}|V_{1:t})$. Importantly, the mapping between actions and object state transitions is neither injective nor surjective. Multiple actions may induce the same state transition (e.g., “bend wire” and “squeeze sponge” both result in “deform”), while the same action primitive may correspond to different object state transitions depending on the manipulated object and contextual environment (e.g., “open microwave” → activate, “open box” → deposit). Therefore, anticipating future object transformations cannot be trivially reduced to anticipating future actions. Rather than predicting what the human will do next, OSCA focuses on forecasting how the physical environment will evolve. This distinction positions OSCA closer to future world-state forecasting and physical environment reasoning, which are important capabilities for embodied AI systems, robotics, augmented reality assistants, and predictive scene understanding.

**Figure 2:**
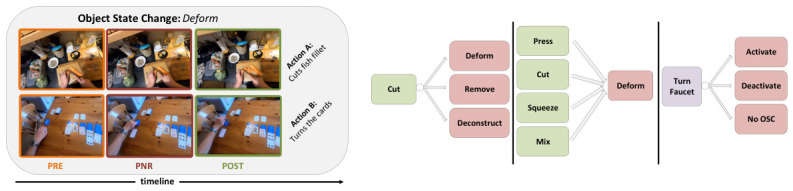
(**Left**) Examples of two different actions that both result the same object state change “deform”. (**Right**) The complex relationship between verbs/actions/object state changes: a verb may imply various state changes; different verbs may indicate the same state change; one action may result in multiple state changes. Raw images are acquired from the Ego4D database.

The OSCA task differs significantly from action anticipation, as it focuses on predicting imminent changes in the object’s state, if any, without requiring the prediction of the verb and possibly the noun categories associated with the anticipated state-modifying action. A state-modifying action may apply to various objects and contexts, resulting in a range of possible state changes, as shown in Figure 2. Also, while it might seem that, given a prediction for the next action, determining the next object state change would be straightforward, estimating such predictions in instructional videos is challenging, as current benchmarks suggest (https://eval.ai/web/challenges/challenge-page/1623/leaderboard/3910/Overall, accessed on 25 May 2026).

The introduction of the newly defined OSCA task opens new avenues for real-world applications across industrial, assistive, and intelligent environments that rely on proactive AI. In manufacturing and assembly, OSCA can facilitate predictive quality control and seamless human–robot collaboration; by anticipating forthcoming object transformations—such as a component being tightened or a part being assembled—monitoring systems and robotic assistants can intervene to proactively prepare the next tool or halt a potentially hazardous action. In the realm of augmented and mixed reality (AR/MR), OSCA could power context-aware guidance and skill-training systems that predict the next procedural steps and visualize expected object changes to assist human operators. Furthermore, integrating OSCA into embodied AI such as Vision–Language–Action (VLA) models [16] or reinforcement learning pipelines endows embodied agents with anticipatory reasoning capabilities related to object transformations and state of the environment. This foresight is critical to improve robotic manipulation, proactive task planning, and human–robot interaction fluency. Beyond industrial settings, this capability holds potential for healthcare and assistive technologies, enabling proactive cognitive assistance for elderly users and enhanced rehabilitation monitoring. Finally, coupling OSCA with generative simulations or digital twins can unlock new frontiers in predictive safety monitoring and explainable forecasting within complex procedural workflows.

To address the new OSCA task, we propose a new formulation that leverages the textual history of past actions and object states, combined with recent visual context, to model the historical dynamics leading up to the decision point. The model can also predict that no state change will occur, indicating a non-state-modifying upcoming action. We evaluate the proposed approach toward the newly proposed OSCA problem to establish baseline results for automated change state anticipation in long instructional videos. We build on the large-scale and challenging Ego4D dataset [10] (Hand & Object Interactions benchmark (https://ego4d-data.org/docs/benchmarks/hands-and-objects/, accessed on 25 May 2026)) which provides egocentric videos by augmenting the available annotation data with object state change labels. This results in Ego4D-OSCA, a variant of the Ego4D dataset that is available to the community. Thus, the main contributions are:

- The introduction of the new problem of anticipating an object state change (OSCA) that will occur in the next, yet unseen, action in instructional videos.
- The introduction of the Ego4D-OSCA dataset, a new benchmark for evaluating solutions to the OSCA problem based on a subset of the Ego4D video dataset. The new annotated data set is already publicly available for review (https://github.com/FORTH-ICS-CVRL-HCCV/Ego4D-OSCA, accessed on 25 May 2026).
- The proposal of the first approach to tackle OSCA, which integrates visual and language features to model the history of performed actions, object states, and their changes. We also present the initial baseline results.

## 2. Related Work

Object states capture dynamic aspects of appearance and/or functionality and are subject to visually perceivable changes, as a result of state-modifying actions [17]. They are also known as object fluents related to changeable object attributes [18,19]. Since there is no prior work on OSCA, we review the literature on closely related topics.

### 2.1. Object State Classification/Recognition

**Object State Classification in Images**: Object states are typically considered as a special subset of “visual attributes”, i.e., visual concepts that are related to the physical and functional properties of objects [8]. Object states and their changes are perceivable by humans and should be perceivable by AI-enabled agents [17]. The majority of the attribute classification approaches follow a similar approach to that of object classification by training a convolutional neural network with discriminative classifiers on annotated image datasets [20]. Few works focus explicitly on state classification [9], while most of them rely on the same assumptions used for the attribute classification task. A prominent direction to tackle this task refers to zero-shot learning. It gained considerable attention in recent years due to its practical significance in real-world applications, mitigating the problem of collecting and learning training data for a very large number of object classes [21]. One such prevalent approach involves the utilization of semantic embeddings to represent objects and their attributes in a low-dimensional space [22]. The work in [23] leverages Knowledge Graphs (KGs) and semantic knowledge in the context of zero-shot object classification. In a similar vein, the work in [24] combines KGs and Large Language Models (LLMs) to address object-agnostic state classification. A recent study by [12] focuses on object state recognition based on the compositional generation of novel object-state images, while the method in [13] introduced a novel conditioned diffusion model that focuses on generating temporally consistent and physically plausible images of actions and object state transformations based on an input image and a text prompt describing the targeted transformation.

An interesting line of work in Compositional Zero-Shot Learning (CZSL) [25] focuses on recognizing previously unseen combinations of known objects and their states in images, using a two-stage recognition strategy. This method leverages both visual and textual features to independently represent object and state attributes, addressing the challenge of disentangling these components from their compositions. A key contribution is a novel gradient-regularized loss term that enhances the separation of object and state information within the visual feature space, ultimately improving the recognition of unseen object–state compositions.

Another related approach [26] employs a Graph Convolutional Network (GCN) with two distinct branches dedicated to object and state prediction, enabling the model to capture complex dependencies between the visual cues associated with each component. The authors further introduce a cross-layer knowledge-sharing mechanism designed to reduce ambiguity when learning state features, which tend to be context-dependent and vary across different object–state combinations.

More recently, PHIER [27] provides a method to improve few-shot and zero-shot state classification by encoding hierarchical relationships between predicates. The approach is based on object-centric scene encoders and a hyperbolic embedding space for predicates and enables generalization across unseen object-state combinations.

**Object State Change Estimation & Action Recognition in Videos**: Object state changes have been considered a meaningful information source in video-based human action understanding and recognition (HAR). In HAR, object state changes are often considered complementary attributes to the visual representation of actions. These changes are typically derived within the visual domain through the utilization of explicit models for object detection and state estimation [14,18,28], or indirect modeling of object states based on general scene changes resulting from action execution [19,29,30]. Several methods exploit object states implicitly to estimate the type of action performed. The work in [19] was among the first to propose a method to automatically discover object states and the associated manipulation actions from videos by leveraging a discriminative clustering framework that jointly models the temporal order of object states and manipulation actions. The work in [18] explored the recognition of object fluents (changeable attributes of objects) and tasks (goal-oriented human activities) in egocentric videos using a hierarchical model that represents tasks as concurrent and sequential object fluents. Moreover, the Attend-and-Interact approach [31] focuses on understanding human actions within videos by analyzing complex interactions across multiple interrelated objects by recognizing different state changes of these objects. In [14,32] a multi-task self-supervised framework is proposed that allows the temporal localization of object state changes and state-modifying actions in uncurated web videos.

Another related proposed framework [33] can recognize object-centric actions by relying only on the initial and final object states. The model can also generalize across unseen objects and different video datasets. The method proposed in [34] aims at disentangling visual embeddings that distinctly represent object states alongside identities, enabling effective recognition and generation of novel object-state compositions through a compositional learning framework. Furthermore, the InternVideo [11] video foundation model was adapted to tackle the tasks of object state change classification and action anticipation in the context of the Ego4D Challenges.

More recently, the VidOSC framework [35] introduced a novel approach for understanding object state changes by segmenting the object parts responsible for these changes in videos, adopting an open-world perspective. Building on this direction, VCI-OSC [36] was proposed as a variational causal inference-based reasoning method that models Object State Changes (OSCs) in videos as a multi-label, frame-by-frame classification task. Their approach leverages a Structural Causal Model (SCM) to accurately localize state transitions within temporal sequences.

A closely related line of work is MOSCATO [37], a benchmark and framework designed to predict the evolving states of multiple objects in long procedural videos, directly addressing the challenge of object state change estimation. The authors also introduce a weakly supervised formulation for multi-object state prediction, explicitly modeling correlations between actions and object states. This strategy improves performance on the Multiple Object State Prediction (MOSP) task, while requiring only action labels during training rather than expensive frame-level object-state annotations.

Recent work studies object state changes from complementary angles, including spatially-resolved change modeling [38], transformation-aware tracking and change understanding [39], and representing procedures explicitly via state transitions [40]. Alongside egocentric advances in multi-modal pretraining [41], hierarchical procedural structure [42], and long-video memory [43], our work differs by focusing on anticipating the next object state change at action onset (rather than detecting/segmenting/tracking it after it happens), leveraging current visual evidence together with structured action/state history.

### 2.2. Action & Next-Active Object Anticipation

Action anticipation involves predicting the label of an action that is expected to occur in the future but has not yet been started/observed [44,45]. This challenge has been studied in both egocentric [10,46] and exocentric [47,48] videos, with the latter becoming increasingly popular in recent years. Short-term action anticipation [49,50,51] focuses on predicting actions in the immediate future. In contrast, long-term action anticipation [52,53] extends to predicting actions or events over a longer period, ranging from several seconds to minutes.

In the context of human–object interactions in videos, active objects [54] and next-active objects [55] refer to specific items that are involved in ongoing or anticipated actions. The active object is the item that a person is currently interacting with within the video. In contrast, the next-active object is the item predicted to be used in the near future, based on the current interaction [56,57,58]. Although not yet in use, it is likely to become involved in subsequent actions. The concept of next-active object anticipation has also been the subject of the short-term anticipation challenge in [10] and is described as the next object to be touched by the user (either with their hands or with a tool) to initiate an interaction. Several methods have been proposed in this challenge for the solution of this problem [51,59,60]. A recent method presented in [61] addresses the challenging problem of segmenting objects that undergo state changes throughout video sequences. This work is particularly relevant to object state change anticipation as it provides a detailed pixel-level understanding of how objects transform over time. Moreover, a recent approach [62] leverages LLMs to understand object state changes through action analysis. This work represents a significant shift toward using language models for visual understanding tasks, demonstrating how LLMs can be employed to reason about object transformations in procedural activities.

More recently, the approach [63], noted as Fiction, introduces a model for 4D future interaction prediction in videos, aiming to anticipate which objects a person will interact with, at which 3D locations, and in what manner. The method fuses past video observations of human actions and environmental context to jointly predict both the “where” (3D location) and “how” (human poses) of future human–object interactions.

Finally, the EgoAnticipator model [64] is proposed for egocentric object–interaction anticipation. This framework predicts upcoming active objects and their associated actions by integrating several components: retentive pre-training for domain-specific representation learning, predictive pre-training to model uncertainty about the future, and mirror distillation to transfer future-aware knowledge. In addition, the model incorporates extended context through long-term memory prompting, allowing it to leverage historical interaction cues when forecasting future behaviors, including anticipated actions and time-to-contact.

Overall, anticipating an object’s state change involves predicting how its condition or form will alter as it becomes involved in an activity, whether currently active or about to become active. This process goes beyond merely identifying which object will be used next (next-active); it focuses on understanding an object’s state evolution during or after its involvement in the interaction. This anticipatory process requires analyzing the current interaction and understanding the transformations that occur as objects are used. Predicting these state changes provides insights into how objects will behave as they become active or next-active, enhancing the ability to interpret event and interaction sequences in video.

## 3. Ego4D-OSCA Dataset

We introduce the Ego4D-OSCA dataset that relies on a subset of the large-scale Ego4D dataset and aims to serve as a benchmark for the assessment of methods for object state change anticipation. The volume and diversity of the Ego4D dataset make Ego4D-OSCA a very challenging dataset for OSCA, as shown in Figure 2. Table 1 provides a high-level comparison between Ego4D-OSCA and existing object state understanding datasets, highlighting its scale, diversity, and unique characteristics.

### 3.1. Dataset Annotation

To enrich the existing Ego4D object state annotations, we integrate object state change annotations based on the following rationale and process. The original Ego4D dataset does not include annotations for the specific object state labels of individual video frames, but rather refers to entire video segments. Additionally, the dataset includes annotations for bounding boxes and object classes across seven critical frames, temporally centered around the PNR of an action, within each video segment. Based on this information, we super-annotate certain critical frames with state-related labels as follows: For each video segment, we annotate the initial and final frames as pre_X and post_X, respectively, where *X* denotes the state change label. The original Ego4D dataset, and hence our derived dataset, contains no instances where an object undergoes more than one state change within a single annotated video segment.

Furthermore, in line with the semantic implications of these changes, we establish three pairs of state changes. Each pair is constructed under the premise that the first action is the inverse of the second concerning the resulting state change. For instance, if *X* and *Y* represent inverse state changes, then the labels pre_X and post_Y are considered samples of identical states. A similar correspondence applies between pre_Y and post_X. For example, the states pre_remove and post_deposit are considered identical, since remove and deposit constitute a pair of inverse state changes. The full set of pre- and post-object state pairs that constitute the target set of object state changes appears in Table 2. There exists a subset of video segments with actions that do not induce state changes, and, therefore, these segments are excluded from annotation. The Ego4D dataset offers eight distinct state change labels: *activate, deactivate, deposit, remove, construct, deconstruct, deform, and other*. However, we contend that there are actions that do not alter the state of an object. To address this, we propose adding a state change category called “No Object-State-Change (No OSC)” to capture actions that do not induce object state change, offering a more comprehensive framework to understand and categorize interactions.

#### 3.1.1. Details on the Annotation Process

**Figure 3:**
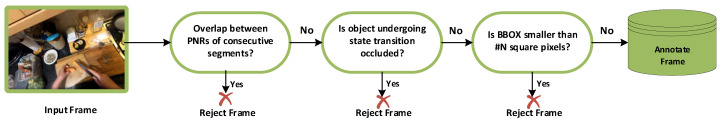
**Annotation pipeline:** Occlusions are checked in the pre- & post-frames. A threshold value for the BBOX area of *N* square pixels (*N* = 100) for each object annotation. Ego4D-SCOD benchmark data are used to automatically annotate the change states per clip.

The annotation process for the state transitions is applied to the pre_Y and post_X frames in each video segment. Overall, the annotation process consists of the following four steps (a schematic representation of the annotation pipeline is shown in Figure 3). First, the PNR moment of the video segment being examined for annotation is compared to the PNR moment of the segment that has been previously annotated. If the PNR of the previously annotated segment is located after the PNR of the segment under examination, then the segment under examination is rejected. The reasoning behind this decision has to do with the learning of the segment features related to state transitions. This alignment of the two PNRs signifies that there is an overlap between the state transition actions of the two segments and therefore the feature learning becomes more challenging. Subsequently, it is examined if the object undergoing state change is occluded. If this is the case, the frame is rejected. Then, the bounding box area of the object is evaluated, and if it is below 100 square pixels—a threshold empirically chosen and commonly used in annotation tools like Voxel-51—the frame is discarded. Finally, the frame passing all checks is annotated with the state label for its transition action.

#### 3.1.2. On the Super-Annotation of Object State Change Classes

**Figure 4:**
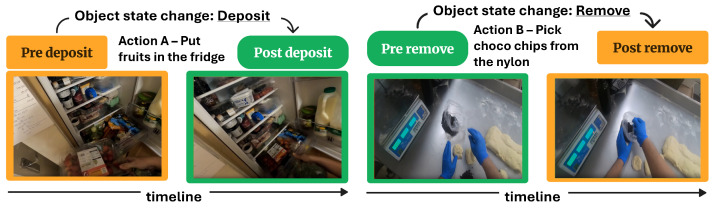
Example of inverse relation for two state-change classes in Ego4D-OSCA (’deposit’ and ’remove’): pre-deposit matches post-remove, pre-remove matches post-deposit. Raw images acquired from the Ego4D database.

As stated in the main paper, the original Ego4D dataset does not provide specific state labels for individual video frames; instead, it offers annotations on state changes tied to entire video segments. These annotations include object bounding boxes and classes for seven key frames, centered around the moment of state change in each segment. Building on this, we augment critical frames with state-related labels. Specifically, for each segment, we label the initial frame as pre_X and the final frame as post_X, where *X* represents the state change. To capture the semantics of these state transitions, we define three pairs of inverse state changes (Figure 4). Each pair reflects that one action reverses the outcome of the other. The conceptualization of these inverse relationships is grounded in the established video understanding literature. Specifically, certain physical actions are *equivariant*, which entails that the temporal reversal of an action produces a visually and semantically realistic instance of its inverse action [68]. We extend this temporal symmetry directly to the physical states of objects. Because of this symmetry, if *X* and *Y* represent inverse state changes, then pre_X and post_Y are considered equivalent, as are pre_Y and post_X. A practical example of this is the pair pre_remove and post_deposit, since “remove” and “deposit” are inverse actions. Table 2 outlines these super-annotated state labels and their inverse association.

**Figure 5:**
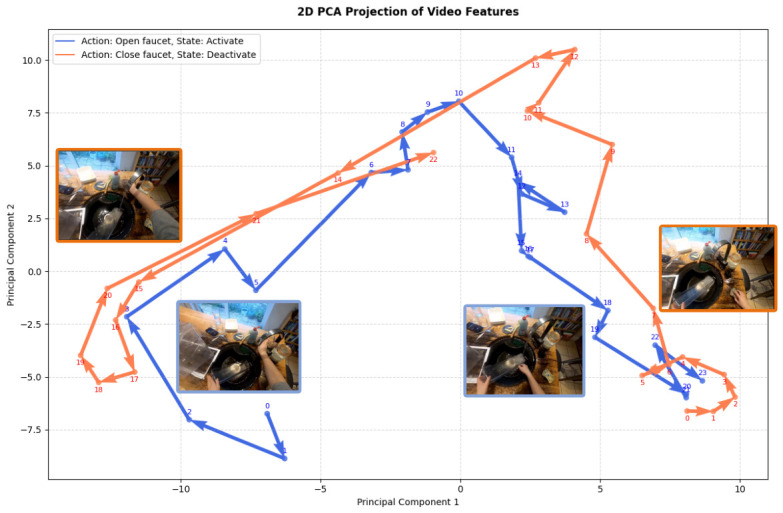
Two-dimensional PCA projection of framewise Ego4D SlowFast feature vectors illustrating the structural and temporal dynamics of opposite object states within a continuous video sequence. The blue trajectory tracks the “open faucet” clip labeled as *activate*, while the orange trajectory tracks the subsequent “close faucet” clip, labeled as *deactivate*.

Additionally, to provide rigorous empirical evidence that the visual representations natively capture this theoretical symmetry, we projected the high-dimensional framewise SlowFast feature vectors from the Ego4D dataset into a 2D trajectory space using PCA (Figure 5). We analyzed two consecutive clips depicting opposite physical transformations: an “open faucet” clip (*activate*) and a “close faucet” clip (*deactivate*). As observed in the low-dimensional projection, both clips occupy a highly localized, intersecting region of the latent feature manifold, capturing the shared object semantics and hand-interaction context. Furthermore, the framewise feature trajectories exhibit a symmetrical, opposing directional flow across time. Crucially, the terminal phase of the *activate* sequence clusters in exact proximity to the initial phase of the *deactivate* sequence, and vice versa. This empirical layout confirms that the baseline visual representations naturally inherit an inverse temporal ordering during opposite manipulation workflows, mathematically validating our structured inclusion of inverse state relations within the Ego4D-OSCA benchmark.

**Figure 6:**
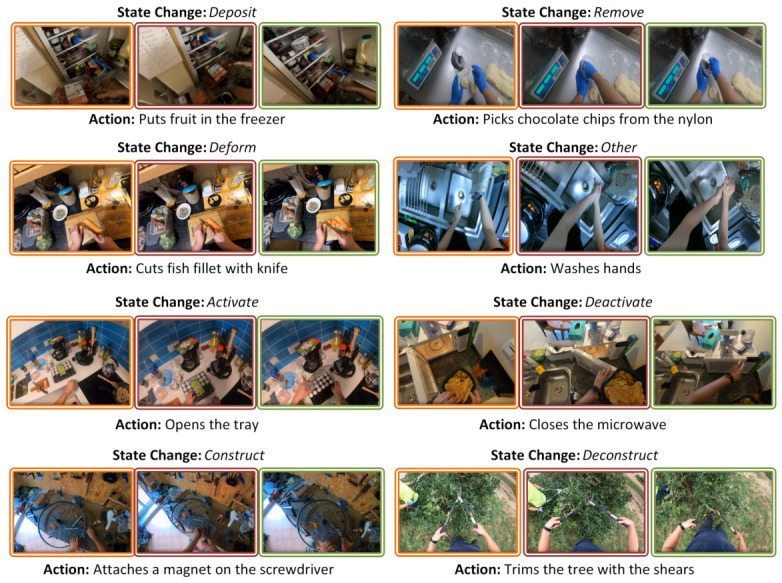
Sample frames of the Ego4D-OSCA dataset depict the initial state, the PNR frame, and the final state for the eight object state change classes (the class ‘No object state change’ is not included). The variability of visual environments/contexts, actions, and objects associated with the object state changes classes is highlighted. Raw images are acquired from the Ego4D database.

**Figure 7:**
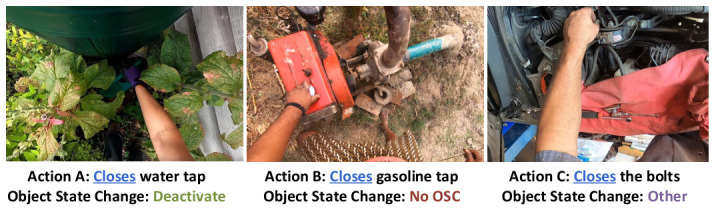
Sample frames from 3 instances of the “close” action, each involving different contexts and objects from the Ego4D-OSCA dataset, each resulting in various types of state changes. Raw images are acquired from the Ego4D database.

Furthermore, to emphasize the complexity and context variability of state change classes, Figure 6 shows sample images from the Ego-OSCA dataset depicting the object state change visual progression. In these few examples, one may notice the inverse association between different state change stages, as well as the large variability of visual environments and contexts, actions, and objects involved in different classes of object state changes. Finally, as also shown in Figure 2, it is worth mentioning that motion motifs (as defined by verb primitives) do not necessarily have a one-to-one correspondence with states. As an example, in Figure 7 we observe that the verb “close” can result in more than one object state change class. This highlights the fact that the object-related context is as important as the motion motif when defining an action, as well as when estimating the anticipated object state change due to the execution of the action. Therefore, to address the OSCA task, an ideal method should build upon the past and current estimates of object detection and state estimation, as well as action recognition methods, to robustly estimate the anticipated state of an object in procedural activities.

### 3.2. Ego4D-OSCA Dataset Statistics

**Figure 8:**
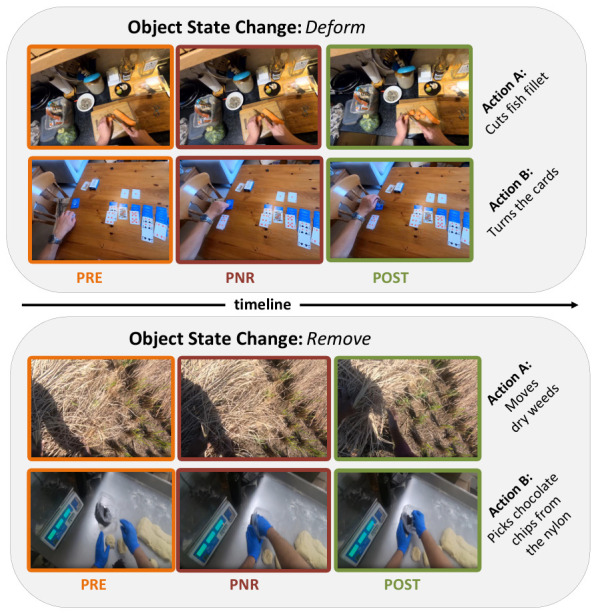
Examples of modifying actions from the “deform” and “remove” object state change classes represented by a triplet of frames (pre-state, PNR, post-state). Each state change is associated with various actions occurring in diverse environments/scenarios, emphasizing the complexity and challenges introduced in the OSCA problem. Raw images are acquired from the Ego4D database.

**Figure 9:**
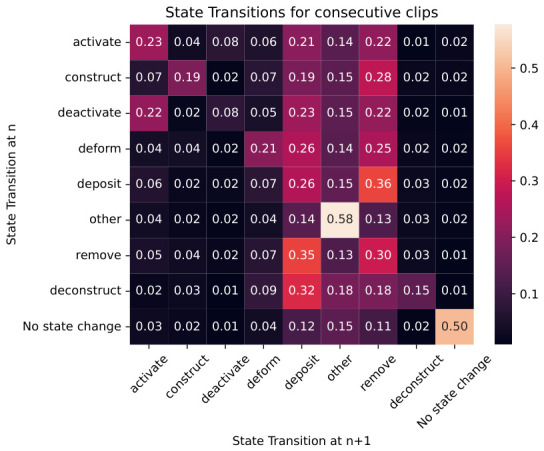
Transition frequencies for all pairs of subsequent object state changes in the Ego4D-OSCA dataset videos.

**Figure A2:**
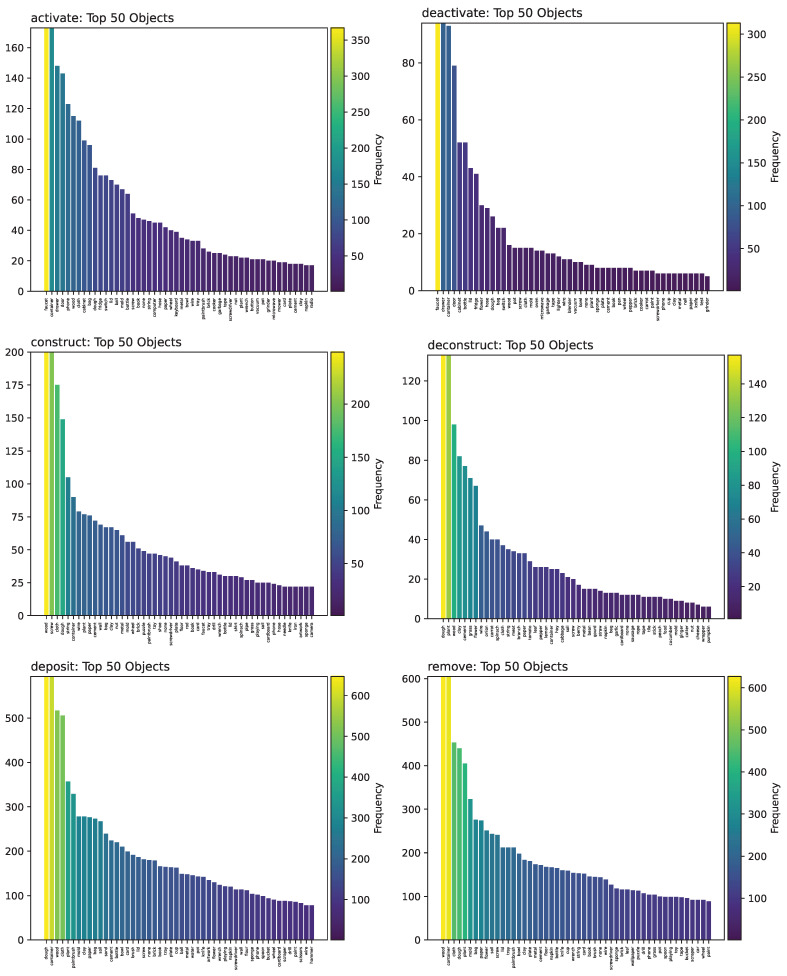
The frequency distribution of the top 50 objects (occurrences of object classes based on the dataset action segments) concerning an object state change class is illustrated in each histogram for the classes ‘activate’, ‘deactivate’, ‘construct’, ‘deconstruct’, ‘deposit’, ‘remove’.

To further characterize the proposed dataset, we analyze the relationships between object states, action verbs, object classes, and activity dynamics. Specifically, we examine the associations between action verbs and object states, the variability of object states across different actions (Figure 8), and the distribution of state transitions throughout activities. These analyses highlight the context-dependent nature of object state changes and the diversity of interactions captured in Ego4D-OSCA, emphasizing the challenges for action and activity recognition and anticipation, as well as object state classification and anticipation. A thorough analysis of the dataset statistics and a detailed discussion are provided in the Appendix A.1 and shown in Figure 9 and Figure A2.

#### 3.2.1. Variability in State Changes & Activity Duration

We observe significant variability in both the number of state transitions and the temporal duration of activities across the *Ego4D-OSCA* dataset [10]. Analyzing the probabilistic dependencies between consecutive actions, as shown in Figure 9, reveals clear temporal ordering and causality—such as “activate” states frequently leading to “deposit” or “remove” states. These highly dynamic and logical transition patterns underscore the necessity for predictive models to incorporate extensive action and state histories.

#### 3.2.2. Dataset Limitations and Generalization Issues

The proposed Ego4D-OSCA dataset inherits limitations from the original Ego4D collection, including class imbalance and bias toward indoor daily activities, which skewed the content toward domestic and low-mobility scenarios. Other issues that may introduce further biases that limit the dataset’s generalization with regard to the diversity of recording devices and annotation process, as crowdsourced narrations and annotation labels due to cultural and linguistic backgrounds may introduce biases in how interactions and object states are described or segmented. These factors may cause models trained on Ego4D or Ego4D-OSCA to overfit to frequent interaction patterns and struggle with rare or context-specific actions, thus affecting generalization to rare actions or industrial scenarios. Minor biases may also arise from heterogeneous capture devices and culturally influenced annotations. Nevertheless, Ego4D-OSCA constitutes the first large-scale benchmark focused on object state change anticipation, providing a valuable foundation for developing and evaluating models in procedural human–object interaction tasks.

### 3.3. Comparison of Datasets Related to Object State Changes

The proposed *Ego4D-OSCA* dataset is compiled using a subset of the popular, large-scale Ego4D v2 dataset [10] comprising egocentric videos for a large variety of human daily living or work activities.

In Table 3 we compare the proposed *Ego4D-OSCA* dataset with existing image and video datasets that also provide annotations related to object states. The target tasks performed using each of the datasets are also noted. In the following, we analyze the most similar ones and argue for the necessity to compile a new dataset as a benchmark for the introduced task of Object State Anticipation in videos.

Ego4D [10]: The Ego4D dataset is among the largest to date, encompassing an extensive collection of videos captured in a wide variety of environments. A subset of this dataset has been utilized for object state detection and classification, as shown in the second-to-last row of Table 3. This subset consists of 92,864 short videos, each featuring a single state-modifying action.

The Ego4D dataset addresses three key challenges related to understanding visual object state changes. Firstly, the Ego4D SCOD (State Change Object Detection) challenge (Ego4D State Change Object Detection Challenge, https://eval.ai/web/challenges/challenge-page/1632/overview, accessed on 25 May 2026) focuses on the bounding box-based detection of the object that undergoes a state change in an action segment (short video clip). The State Change Classification task is defined as the multi-class classification of object state changes in a video clip where a state-modifying action occurs, for example, identifying that the state of a cup has changed from filled to empty. Second, the binary state change classification variant is realized as an Ego4D challenge (Ego4D Object State Change Classification Challenge, https://eval.ai/web/challenges/challenge-page/1627/overview, accessed on 25 May 2026) to detect whether a state change was performed or not in an action segment (video clip). Moreover, the Ego4D State Change Localization challenge (Ego4D Object State Change Temporal Localization Challenge, https://eval.ai/web/challenges/challenge-page/1622/overview, accessed on 25 May 2026) involves pinpointing the exact frames in the video where the state change occurs. Accurate localization is crucial for understanding the precise timing and context of the state transitions within the egocentric video perspective. These challenges are designed to advance the understanding and development of AI models in recognizing and interpreting state changes in dynamic and realistic scenarios captured from a first-person viewpoint. Finally, a series of workshops in major conferences have been organized based on the Ego4D dataset and related tasks/benchmarks, such as 2nd International Ego4D Workshop @ ECCV 2022 (https://ego4d-data.org/workshops/eccv22/, accessed on 25 May 2026), 1st Ego4D Workshop @ CVPR 2022 (https://ego4d-data.org/workshops/cvpr22/, accessed on 25 May 2026) and the Joint 3rd Ego4D and 11th EPIC Workshop on Egocentric Vision @ CVPR2023 (https://cvpr.thecvf.com/virtual/2023/workshop/18537, accessed on 25 May 2026).

Comparison: The Ego4D-OSCA dataset comprises long videos of sequential state-modifying actions that correspond to any of the nine classes of object state changes: *deposit, remove, construct, deconstruct, activate, deactivate, deform, other, and no-state-change*. A distribution of the samples across the 9 object state labels is presented in Table 4. In contrast, the current subsets of Ego4D used for detecting and classifying object states (Ego4D SCOD & OSCC benchmark) comprise short videos, each depicting a single action. This renders the subsets unsuitable for addressing the problem of anticipating object state changes in procedural videos that comprise consecutive actions under the same scenario (activity).

ChangeIt [14]: The ChangeIt dataset comprises unedited videos sourced from YouTube and automatically generated labelling of actions. The designated tasks for analysis on this dataset involve the identification and temporal localization of the initial state, end state, and state-modifying action in a video. A set of 44 state-changing actions is provided, each demonstrated in approximately 15 videos on average. In total, there are 34,428 videos with an average duration of 4.6 min.

Comparison: The newly introduced Ego4D-OSCA dataset comprises sequential videos featuring actions, some of which may involve state changes, while others may not. In contrast, the ChangeIt dataset consists of single-action clips, one object state change is performed per clip. Ego4D-OSCA offers a wide range of scenarios without imposing any limitations and encompasses video durations spanning from minutes to hours. Conversely, the ChangeIt dataset confines scenarios to irreversible actions, aiming to eliminate instances where two actions return an object from an initial state to the same initial state via an intermediate state. Additionally, it excludes videos exceeding 15 min in length.

MOST [62]: The newest dataset related to object states in videos addresses the problem of temporal segmentation of multi-label object states. It includes manually collected instructional videos from YouTube, covering six object categories: apple, egg, flour, shirt, tire, and wire, each with around 10 annotated object states. These states represent common appearances or conditions an object may take. Annotators marked the time intervals when specific object states were visible, resulting in a dataset of 61 fully annotated videos with a total duration of 159.6 min. Unlike other datasets, such as ChangeIt, which focuses only on state transitions, MOST captures diverse object states, even if those are not tied to specific actions, offering a comprehensive benchmark for object state recognition.

Comparison: The MOST dataset is designed to assist the recognition of multiple object states for a *single object category per video*. In contrast, our proposed Ego4D-OSCA dataset focuses on anticipating the *state change class of multiple objects within each video*. This means that while MOST aims to recognize objects’ current state, Ego4D-OSCA emphasizes predicting what an object’s state change will be as a result/effect of the next (near future), yet unobserved, action. Additionally, Ego4D-OSCA covers a larger dataset with 1498 videos and 475 object classes, compared to MOST’s 61 videos and 6 object categories. We see potential in bridging this gap in future work by adding annotations for state change classes to the MOST dataset, which could open up new avenues for research in multi-label object state change anticipation. Additionally, contrary to the proposed Ego4D-OSCA dataset, the MOST dataset does not provide annotations regarding the actions and activities across the video.

Such information, which our proposed methodology utilizes, can be crucial for understanding the progression of actions and their effects on objects, enabling models to predict future states and state changes more accurately. Without this contextual information, it becomes significantly more challenging to infer the relationships between actions, object interactions, and subsequent state changes, limiting the ability to anticipate how an object might evolve within the dynamic environment of a procedural activity.

HowToChange [35]: This dataset is generated using a subset of The Food & Entertaining category of the HowTo100M dataset [69], a large-scale dataset of narrated videos. The reasons for selecting that particular subset are: (1) it constitutes one-third of the entire HowTo100M video collection, (2) cooking tasks within this category provide a rich variety of objects, tools, and state changes, making it an excellent testing ground for open-world Object-State-Change (OSC) understanding, and (3) in cooking activities, a single state transition can often be linked to a diverse array of objects, creating opportunities for compositional learning. The dataset provides annotation data related to three OS classes (initial, transitioning, and end state) related to OSC localization. A total of 498,475 videos and 11,390,287 ASR transcriptions processed with LLAMA2 reveal the most frequently observed state transitions and the associated objects. This information is utilized to establish an OSC vocabulary, identifying 134 objects, 20 state transitions, and 409 unique OSCs.

Comparison: The HowToChange dataset contains clips that involve a single state-changing action that is not compatible with the requirement for subsequent actions in a single video, as in Ego4D-OSCA. On the other hand, it contains novel objects in the test set, which sets the dataset as a challenging benchmark for OSC analysis.

VSCOS [61]: This dataset comprises 1905 video clips of an average duration of 7.4 s, capturing various interactions with objects and state changes. These videos encompass 30 action categories and 124 object categories, resulting in a total of 271 valid combinations. The state changes in the dataset can be categorized into four prominent groups: Rigid Object Composition and Decomposition (e.g., combine, cut, split, disintegrate, unpackage), Non-rigid Object Transformation (e.g., pour (liquid), crack (egg)), Object Appearance Change (e.g., cook, clean), and Object Articulation (e.g., open, close, twist).

Comparison: Each video in the VSCOS dataset contains a single state-changing action. The test set of this dataset is challenging because it encompasses the following cases: novel objects-seen state changes, seen objects-novel state changes, and novel objects-novel state changes.

VOST [66]: VOST is introduced as a benchmark for video object segmentation, emphasizing intricate object transformations. In contrast to current datasets, VOST introduces scenarios where objects undergo processes such as breaking, tearing, and molding, leading to substantial alterations in their overall appearance. Comprising over 700 high-resolution videos captured in diverse environments, each video in the dataset has an average duration of 21 s and is meticulously labeled with instance masks of objects across frames.

Comparison: The VOST dataset comprises videos depicting objects undergoing state-changing transformations. While a change in an object’s state is demonstrated in each clip of the dataset, the state types are not explicitly labeled. According to the authors, the transformations are indicated by 51 specific verbs such as cut, peel, apply, break, open, scoop, fold, mold, etc. This dataset excludes videos with no transformations. In contrast, Ego4D-OSCA consists of sequential videos that exhibit a series of object state changes alongside videos that lack such changes. Ego4D-OSCA encompasses 117 verbs and 475 objects.

## 4. Object State Change Anticipation—Baseline

**Figure 10:**
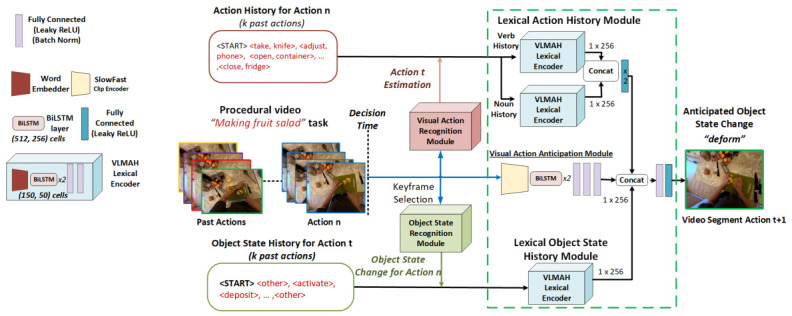
Overview of the proposed baseline framework for the object state change anticipation task. The proposed framework anticipates object state changes by integrating real-time visual data and a historical record of past actions and object state changes. Raw images are acquired from the Ego4D database.

The proposed framework, shown in Figure 10, draws inspiration from the effectiveness of combining visual and lexical information for semantic action/activity encoding. It adopts a three-stream architecture: a visual encoding module captures the visual attributes of ongoing actions, while two lexical encoders extract semantic nuances from a procedural representation of past actions and object states. These representations are fused to anticipate the next object state—i.e., the state the object will assume during the subsequent action. By integrating visual and lexical cues, the framework aims to holistically capture the dynamics and contextual intricacies of object-state transformations across sequential actions.

The design of our framework draws from the recent VLMAH model [70], which was specifically tailored for the task of *action* anticipation. We augment this architecture by introducing specialized object state history encoding modules. Additionally, we redesigned the action history module to facilitate disjoint encoding, capturing both the motion motifs in actions (verbs) and the transitions of objects-in-use (nouns) between actions.

As illustrated in Figure 10, the proposed framework consists of two primary components: (a) the current action and object state estimation module, and (b) the object state anticipation module, depicted within the thin-dotted rectangle. Our contribution resides in (a) the conceptualization of this framework and (b) the development of the object state anticipation module.

**Visual Encoder**: For this module, we employ a lightweight visual encoder consisting of a single-branch bidirectional long short-term memory (BiLSTM) component followed by a multi-layer perceptron (MLP). We selected this simplified design based on the objective of temporally encoding the enduring relationships among encoded short-term segments extracted from the input video while also maintaining a high degree of computational efficiency, which is critical for real-time monitoring on consumer-grade hardware. Our model relies on an external pre-trained human action recognition model, such as SlowFast [71] or TSN [72] to provide encodings of short-term spatio-temporal dependencies between the frames inside a single segment. As shown in [73], the design choices of the VLMAH pipeline, adopted in the proposed baseline as well, enable strong performance in action anticipation tasks while maintaining a substantially lower computational footprint compared to heavier convolutional and transformer-based video architectures.

**Action & State History Encoders**: As illustrated in Figure 10, both encoders exploit the model design of the lexical encoder of the VLMAH model [70], which follows a NLP neural network design consisting of BiLSTM and MLP components. The decision to employ a simple NN for encoding the history, instead of utilizing LLMs was motivated by several factors. The computational efficiency of LLMs such as GPT or LLaMA often entails significant resource requirements for training and inference [74], whereas a simpler neural network architecture mitigates computational overhead. LLMs are pre-trained on general text corpora and may not capture the domain-specific nuances inherent in the textual data related to action histories and object states. The simplicity of the chosen architecture facilitates interpretability, data efficiency, and customization, affording greater control over the model’s behavior and adaptation to the task’s requirements.

**Learning Objective**: The objective for training the model was exclusively focused on evaluating the anticipated state estimate. This objective was formulated using the cross-categorical entropy loss, which is well-suited for multi-class classification tasks, such as predicting object states across different categories.

$$L=-\frac{1}{N}\sum_{i=1}^{N}\sum_{c=1}^{C}y_{i,c}\log\left({\hat{y}}_{i,c}\right),$$

where *N* is the number of samples in the dataset, *C* is number of object state categories, $y_{i,c}$ is the ground truth next state label for the object-in-use in the current action sample *i* and ${\hat{y}}_{i,c}$ is the predicted next state probability.

During training, the proposed framework leverages oracle action and state detectors to provide the action and state history, respectively, for each clip in the dataset (see Figure 10). These detectors estimate the current action and object state observed in the clip, serving as ground truth annotations for training purposes. However, it is important to note that for inference toward real-world applications, there is a requirement for current action and object state recognition models to provide input to the framework. Consequently, our model is solely tasked with the learning objective of next-object anticipation, focusing exclusively on predicting the future state of the object. By decoupling the training and inference phases in this manner, the model can effectively learn the dynamics of object-state transitions without the added complexity of simultaneously predicting the current action.

## 5. Implementation, Experiments and Results

**Implementation Details:** The proposed state anticipation model (dotted rectangle in Figure 10) is trained using the Adam optimizer, a batch size of 32, a learning rate of $1\times 10^{-4}$, without any temporal augmentations (clip or frame cropping). Short-term associations between neighboring segments of an input video are represented using the pre-extracted SlowFast frame-level features from Ego4D. For the selection of the pre- and post-state keyframes, we exploit the PNR annotations of Ego4D per clip, which correspond to the first frame in a clip where the state change/transition is visible.

**Evaluation metrics:** The evaluation of all examined models was conducted using top-1 and top-5 mean accuracy, and F1-score, following standard practices in the relevant literature.

### 5.1. OSCA Results

In Table 5, we compare variants of an object state anticipation model to highlight the impact of incorporating lexical histories of past actions and object states on the anticipation performance. The vision-only model (VID-A) relies solely on the visual representation of the current action. We observe modest performance levels.

**OSCA under ideal action & state recognition**: When ground-truth lexical histories of past actions are introduced through an oracle recognition model (VNLP (O-Action)), a slight performance improvement indicates the potential benefit of contextual action information. Notably, incorporating lexical histories of past object states from an oracle recognition model (VNLP (O-State)) leads to a significant performance gain, which highlights the importance of modeling object state dynamics in anticipation tasks. Further improvements are observed when both lexical histories are integrated into the model (VNLP (O-Action, O-State)), demonstrating the synergistic effect of leveraging contextual information from actions and object states. Overall, the low anticipation scores highlight the inherently challenging nature of the task and the intricacy of the dataset scenarios.

**OSCA under actual action & state recognition**: We conducted experiments where the oracles were replaced with existing baseline models. In this setting, the output of these models populates the action and state histories. We employed: (a) the well-established SlowFast [71] model for action recognition; (b) the object-agnostic state classification method of [9] as the current state classifier, with minor modifications as follows.

*Action recognizer*: For action classification, we fine-tuned the SlowFast model [71] on a subset of Ego4D. We derived the train/validation/test splits from the long-term anticipation training set of Ego4D using a 60/20/20 split. The adaptation of these data to the action recognition task resulted in 5742 action classes and a total of ≈65K video clips (with a mean of ≈10.7 sample clips per action class). SlowFast achieved 12.86% Top-1 and 33.69% Top-5 accuracy for the task of current action recognition. The observed low performance can be attributed to the large number of action classes, limited samples per class, and Ego4D’s inherent motion and appearance similarities across actions.

*Object state recognizer*: For the object-agnostic state history, we adapt the model of [9], which relies on the outputs of two distinct state classifiers. Each classifier receives the first (pre) or the last (post) frame of each video segment as input to predict the object state label for the respective frame. The prediction of the state-change label for the video segment considers both outputs and is derived under the following rules.

If the predicted states are pre_X and post_X, the inferred state change is *X*. If the predictions are pre_X and post_Y, where *X* and *Y* are distinct and inverse, no state change is inferred. Otherwise, the prediction defaults to the second classifier’s output; e.g., post_Y implies a state change of *Y*. For instance, pre_activate and post_activate yield activate, while pre_activate and post_deactivate yield no state change. In other words, since this classifier analyzes individual static images, it cannot directly predict a state change (a transition). To infer the state change for a given video segment, we analyze and compare the predictions made for the corresponding pre- and post-frames. Due to the inherent semantics of the transitions, six of the eight classes form three pairs of inverse transitions (e.g., “Activate” and “Deactivate”). In such cases, the pre-frame of one transition is identical to the post-frame of its inverse. Therefore, if the classifier predicts states pre_X and post_Y for a segment, the inferred state change is X. For the special case where the predictions are pre_X and post_Y (where X and Y are distinct and inverse), no state change is inferred.

This object-agnostic recognizer achieved 25.4% mean state recognition accuracy. We observed a significant drop in accuracy by replacing oracles with realistic recognizers to populate the action and state history buffers in our baseline OSCA model (last block of lines in Table 5). This accuracy difference highlights the crucial role of precise current action and object state recognition in effectively anticipating near-future object states within dynamic environments.

A notable finding concerns the performance gap in Top-1/5 Accuracy between (a) the variant that leverages only past object state history, namely VNLP (O-State) and VNLP (State), in Table 5 and (b) the variant that jointly exploits both past action and object state histories, namely VNLP (O-Action, O-State) and VNLP (Action, State) in Table 5. Given the considerable imbalance across action and object state categories in the proposed Ego4D-OSCA dataset and its source dataset, Ego4D, as well as the 1:M or M:1 relationship of actions toward the state change set (depicted in Figure 7), accuracy alone may not fully reflect model performance. The F1-score, which integrates both precision and recall while remaining less sensitive to the abundance of true negatives from majority classes, provides a more informative measure. Consistent with this, the F1-scores reported in Table 5 suggest that models combining both action and state histories can learn more semantically coherent representations, improving object state anticipation.

**Figure 11:**
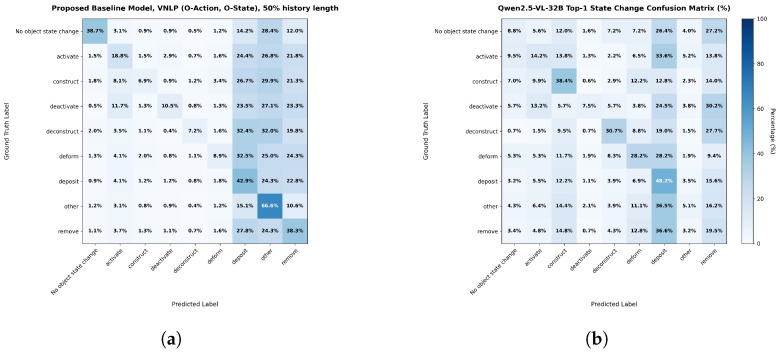
Model evaluation showing the per-class confusion matrix of (**a**) the proposed baseline model, VNLP (O-Action, O-State), 50% history length, as shown in Table 6, and (**b**) the best-performing VLM (Qwen2.5-VL-32B) model.

**Per-Class Error Analysis:** To gain deeper insight into the baseline architecture’s operational boundaries, we evaluate its per-class performance distribution using the detailed confusion matrix of the top-performing configuration (as illustrated in Figure 11). The empirical results highlight a defining algorithmic strength in identifying procedural stability, with the model achieving its highest true positive rate of 66.64% on the *No state change* category. This robust performance demonstrates that the visual–linguistic history successfully captures contextual and environmental indicators signifying non-state-modifying steps.

Conversely, the analysis reveals two prominent failure modes that delineate the system’s current limitations. The first is a systemic majority-class attractor effect heavily driven by the long-tail distribution of the Ego4D-OSCA benchmark. Across nearly all active transition categories, the model exhibits a steady 24–32% error rate where true state modifications are erroneously misclassified as *No state change*. The second vulnerability relates to fine-grained semantic and temporal boundary ambiguity between highly active, opposing interactions. Specifically, true *deactivate* transformations are severely misclassified as *activate* in 32.51% of cases, while true *construct* events are frequently blended with *activate* predictions (27.81%). This cross-class confusion underlines that while the history module tracks overarching procedural structure, it struggles to cleanly separate the subtle temporal directionality of opposite state boundaries during overlapping hand–object interactions, which indicates the challenging nature of the proposed task and the importance of the proposed dataset.

### 5.2. Object State & Action Recognition Impact

To further demonstrate the impact of the performance for current action and object state recognition on the OSCA task, an additional experimental setup was derived where hypothesized recognizers of different accuracy are considered. To this end, we uniformly introduce noise, representing erroneous estimations, to both the action and state histories, to simulate the performance of the respective recognizers.

Table 7 presents the results obtained under three varying levels of label noise (rows 2–4), compared to the scores achieved when ground truth labels are considered (noise level is 0%). The noise levels correspond to the erroneous estimate rate generated by the recognizers, i.e., 25% corresponds to a recognizer with 75% mAcc. We observe that the state anticipation performance is influenced by the recognizer’s accuracy, demonstrating an approximate 4–5% reduction in OSCA accuracy for every 25% decrease in object state and action recognition accuracy. Notably, despite substantial performance loss in state and action recognition, the anticipation model exhibits only a marginal performance loss. This finding can be attributed to the compensatory capability of the visual component of the model, which effectively accommodates dynamic and previously unseen sequences of action and state histories.

### 5.3. Object State & Action History Length Impact

To explore the influence of history length on the prediction of the next object state, we analyzed how varying temporal context sizes affect model performance. Specifically, we evaluate the baseline model under two distinct experimental paradigms: first, through a symmetrical reduction where both action and state history lengths are compressed equally, and second, through an asymmetrical modification where the history window for actions and states is decoupled and varied independently. By progressively truncating these tracking windows from the maximum length down to minimal local context frames, we aim to isolate the explicit long-range temporal dependencies and evaluate the relative informational density provided by sequential action tokens versus preceding object states.

**Symmetrical History Truncation:** The empirical evaluation of temporal window lengths presents a non-linear relationship between historical context retention and next object state prediction performance. As shown in Table 6, maintaining the full history sequence length (100%, matching 1445 tokens), i.e., past actions and past object states, establishes the upper bound for macro structural stability. However, it underperforms in raw classification power compared to tighter sequence windows. Peak predictive accuracy is observed when temporal history is halved (50%, 722 tokens), achieving the highest accuracy. This optimization point indicates that while macro pattern cohesion benefits from maximum context lengths, overly extended sequence windows introduce optimization bottlenecks or ambient noise that obscure immediate causal dynamics. This is highly aligned with the unconstrained, long-form nature of the Ego4D dataset, where daily egocentric activities are characterized by frequent sub-task transitions, environmental distractions, and procedural interruptions. Consequently, keeping the entire historical sequence forces the model to process distant, out-of-context actions that degrade local prediction clarity. Conversely, truncation down to extreme minimums breaks this balance, resulting in a distinct drop in accuracy, verifying that a foundational baseline of local tracking is required to handle state evolution accurately against rapid egocentric viewpoint shifts and immediate object manipulations.

**Asymmetrical History Truncation:** Decoupling the individual contributions of action tracks and physical state queues highlights an asymmetric multi-modal reliance during state-transition reasoning. As detailed in Table 8, the bottom configuration baseline underscores that the next object state prediction is fundamentally tied to historical state trajectories over sequential action intents. Stripping away state history entirely (100% Action/0% State) causes a severe performance degradation. Within the context of egocentric video like Ego4D, this collapse occurs because first-person action labels are inherently complex, highly varied, and prone to visual ambiguity or occlusion caused by hand-object interactions. Without an explicit history of the target object’s state trajectory to ground the sequence, action tracking alone cannot reconstruct the causal path. Conversely, relying exclusively on state trends (0% Action/100% State) maintains a resilient performance profile, demonstrating that the progressive evolution of an object’s physical states provides a strong structural prior for daily procedural tasks. Interestingly, introducing minor action histories provides a strong regularizing effect on the state landscape. When the context lengths are set unevenly, keeping a higher ratio of historical state trends over action tokens (25% Action/50% State) maximizes predictive clarity. This trend proves that within Ego4D’s unconstrained workflows, object state history supplies the target sequence structure, while action streams act as filtering constraints to filter out secondary motions and sharpen boundary predictions.

### 5.4. Comparison with Vision-Language Models (VLMs)

We benchmarked several top-performing, open-source Vision-Language Models (VLMs) to thoroughly evaluate the difficulty of the OSCA task and contextualize our approach against recent advancements in foundation models. The comparative results are summarized in Table 9, while the prompt, details on the characteristics of VLMs and the inference setup of the experimental evaluation are provided in Appendix A.3 and Appendix A.4 and Table A1). Among the evaluated foundation models, the Qwen2.5-VL architecture demonstrated the most competitive performance. Specifically, Qwen2.5-VL-32B achieved the highest VLM performance across both Top-1 accuracy (23.09%) and Macro F1-score (18.03%). InternVL2.5-38B followed closely with a Top-1 accuracy of 22.54% and an F1-score of 16.48%. Conversely, other models like LLaVA-NeXT-Video-34B struggled significantly, yielding a F1-score of just 11.73%. Crucially, when comparing these results to our proposed baseline, we observe that our lightweight, history-conditioned framework (*VNLP (Action, State)*) significantly outperforms the best available VLM. Our baseline achieves a Top-1 accuracy of 29.42% and a F1-score of 26.29%, thereby outperforming Qwen2.5-VL-32B’s F1-score by an absolute margin of over 8%. When assuming ideal recognition conditions (*VNLP (O-Action, O-State)*), this performance ceiling scales up to a 37.12% F1-score, completely eclipsing the foundation models.

This substantial margin in F1-score is driven by the VLM’s inability to resolve visual ambiguity without historical context, which leads to scattered, off-diagonal errors rather than a simple majority-class bias. A direct comparison of the confusion matrices (Figure 11) reveals concrete examples of this structural deficit. First, the VLM severely struggles to identify procedural stability, i.e., cases where an action occurs but does not physically alter the object’s state. Qwen2.5-VL-32B correctly predicts “No object state change” in only 8.8% of cases, frequently predicting erroneously active transformations such as “deposit” (26.4%) or “remove” (27.2%). In contrast, our history-conditioned baseline accurately identifies this passive state 66.6% of the time. This demonstrates that tracking past states is necessary to know when an object’s physical evolution has temporarily paused. Second, without a memory of preceding steps, the VLM cannot reliably disambiguate visually similar actions. For example, when the ground truth next state is “deactivate”, Qwen guesses the exact opposite transition (“activate”) 13.2% of the time, while scattering another 54.7% of its predictions into unrelated classes like “deposit” and “remove”. Furthermore, the baseline vastly outperforms the VLM on the highly ambiguous “other” category (66.6% accuracy versus Qwen’s 5.1%). By explicitly conditioning the prediction space on the procedural history, our framework inherently constrains the allowable state transitions. It effectively filters out temporally illogical hypotheses (e.g., predicting an object will be “activated” when the history indicates it already is), resulting in fewer scattered errors and a higher overall F1-score.

## 6. Discussion on Future Work & Applications

While OSCA provides a strong foundation for object state change anticipation in egocentric videos, there remain multiple directions to advance both the benchmark and the broader research area. Below, we outline key avenues for future work and potential uses of the dataset that could inspire follow-up studies.

Starting from the architectural design that the proposed baseline is built upon, several improvements could be made to its components. The *action recognition* module, which currently relies on a SlowFast backbone, could benefit from stronger temporal reasoning and richer contextual integration. Replacing or augmenting this component with transformer-based video architectures such as Video Swin [80] or MViT [81] could enhance the representation of long-range dependencies and fine-grained motion patterns. Incorporating object- and hand-centric cues, either through explicit interaction modeling or by conditioning features on detected object trajectories, would allow the recognizer to focus on the elements most relevant to object state transitions. Furthermore, multimodal fusion strategies that integrate complementary depth [82], audio [83] or textual signals [84] at the current action level could help the model disambiguate visually similar actions and improve robustness under challenging conditions. Concerning the *object state recognition module*, our goal is to explore the capabilities of Siamese architectures [85]. Specifically, instead of using separate classifiers for the pre- and post-frames, a single Siamese network can be utilized that takes both frames as input and directly outputs the corresponding state change. This approach is expected to yield a more refined classifier with improved accuracy.

Regarding the *history derivation*, large language models (LLMs) [86] and vision–language models (VLMs) [87,88] offer a promising direction for enriching temporal reasoning across all components of the proposed baseline. These models could be employed to populate and maintain structured histories of past actions and object states, serving as semantic memory modules that encode long-term dependencies beyond the temporal window of the current visual input. In practice, a VLM could summarize past frames into symbolic descriptions of key actions, object interactions, and intermediate states. At the same time, an LLM could reason over these summaries to infer causal and procedural dependencies. The derived history representations could then be queried or fused with the current action recognizer (e.g., SlowFast or its transformer-based successor) to provide contextual priors about likely ongoing actions, and collaborate with the state estimator to constrain the space of plausible object states given recent manipulation patterns. The state change anticipation module could then integrate these history-aware embeddings to reason about temporal consistency and anticipate future transitions more accurately. In this way, LLMs and VLMs could act as complementary reasoning agents, enhancing both the causal interpretability and temporal coherence of OSCA systems.

Finally, from a training perspective, joint or multi-task optimization [14,89], where action recognition, object state estimation, and anticipation are learned together, may lead to more semantically aligned representations that better capture causal relationships between actions and their resulting state changes.

Beyond improvements to the current baseline, several methodological directions could advance OSCA systems more broadly. In particular, richer context modeling represents a key avenue for future research, enabling frameworks to go beyond reasoning about a single target object in isolation. Multi-object reasoning could capture complex scenarios where state changes are interdependent, such as when a tool’s condition directly affects the transformation of another object. To this end, dynamic scene graph representations [90] offer a structured way to track evolving object–object and object–action relationships, while temporal abstraction mechanisms can incorporate knowledge of high-level task stages. Furthermore, drawing architectural inspiration from the multi-object tracking domain is highly beneficial [50,91,92]; egocentric video sequences frequently exhibit sudden viewpoint variations, rapid camera movement, and severe object deformations that mirror the exact spatial–temporal context modeling challenges tackled by advanced target trackers and data association techniques. Integrating these advanced spatial–temporal tracking modules with modern egocentric tracking paradigms for understanding human–object interaction will enable future models to leverage long-horizon dependencies better, e.g., by reducing errors in cases where the upcoming state change is heavily influenced by actions performed much earlier in the procedure or occluded by human hands or other interacting objects.

Another promising direction is expanding the benchmark to evaluate OSCA under broader conditions and in specialized domains. While Ego4D-OSCA provides a strong starting point, incorporating third-person instructional datasets such as Assembly101 [47] or IndustReal [93] would enable studies on viewpoint robustness and domain transfer. Synthetic datasets generated through simulation platforms, such as NVIDIA’s Omniverse platform or video generation models [13], could fill gaps in coverage for rare or safety-critical state changes. Meanwhile, controlled variations in simulated environments could support targeted model stress testing. Bridging synthetic and real data through domain adaptation would be especially valuable for preparing OSCA systems for deployment in real-world applications. In particular, incorporating object state recognition and anticipation into industrial settings, such as assembly lines, quality control, or predictive maintenance, could enable early detection of process deviations, automated verification of assembly steps, and proactive intervention before costly errors occur, ultimately improving efficiency, safety, and product quality.

Finally, human-interpretable OSCA presents an important step toward practical adoption. Future models could generate natural-language explanations [94,95] that connect predicted state changes to observed actions, object affordances, and contextual cues, fostering user trust. Interactive querying would allow operators to ask about the anticipated future of specific objects, supporting proactive decision-making in procedural tasks. Additionally, incorporating counterfactual reasoning [96], i.e., predicting how object states would differ if alternative actions were taken, could expand OSCA’s role from passive prediction to active guidance, making it a valuable tool for training, error prevention, and collaborative human–AI task execution.

## 7. Conclusions

This paper introduces the task of object state change anticipation (OSCA) during procedural activities, proposing a framework that integrates lexical histories with recent visual context to improve anticipation accuracy substantially. To support this task, we curated Ego4D-OSCA, a specialized subset of Ego4D featuring new, fine-grained annotations of object state changes, which enable reproducible evaluation and foster community engagement. Together with the future research directions outlined above, our contributions establish a foundation for advancing OSCA toward practical applications in domains such as industrial assembly, healthcare, and augmented reality. We hope that Ego4D-OSCA will serve not only as a benchmark for state change anticipation, but also as a springboard for innovations in interpretable, proactive, and trustworthy human–AI collaboration.

## Figures and Tables

**Table 1:** Comparison with other image and video datasets that contain annotations related to object state changes. * Modalities indicate available data sources for object state change tasks.

Datasets	Modalities *	OSC-Related Task	Actions/Video	Samples	Obj. State Classes	Actions	Objects
Fire et al. [65]	Video	Detection	Single	490	17	–	13
ChangeIt [14]	Video	Temporal Localization	Single	34,428	–	44	–
HowToChange [35]	Video & Text	Temporal Localization	Single	498,475	20	–	134
VSCOS [61]	Video	Segmentation	Single	1905	4	271	124
VOST [66]	Video	Segmentation	Single	713	–	–	155
Ego4D [10]	Video & Text	Detection & Classification	Single	92,864	8	–	478
OSCaR [67]	Video & Text	Detection & Classification	Single	14,084	–	–	1000
ChopNLearn [12]	Video & Text	Compositional Image Generation	Single	1260	8	1	20
**Ego4D-OSCA (Ours)**	Video & Text	**Anticipation**	**Multiple**	**1498**	**9**	**5742**	**475**

**Table 2:** Super-annotated state change actions and their corresponding pre-/post-state labels for each video segment where the state-modifying action occurs. Pairs such as activate–deactivate, deposit–remove, and construct–deconstruct represent inverse object state change actions. Frame-level state labels corresponding to the same state are shown with the same color.

OSC Action	Activate	Deactivate	Deposit	Remove	Construct	Deconstruct	Deform	Other
Pre-State	pre	pre	pre	pre	pre	pre	pre	pre
label	(activate)	(deactivate)	(deposit)	(remove)	(construct)	(deconstruct)	(deform)	(other)
Post-State	post	post	post	post	post	post	post	post
label	(activate)	(deactivate)	(deposit)	(remove)	(construct)	(deconstruct)	(deform)	(other)

**Table 3:** Comparison with other image and video datasets that contain annotations related to object state changes. In *Ego4D-OSCA*, a sample refers to a video of an entire activity, which might consist of multiple actions.

Dataset	Modalities	Task	Year	Actions/Video	Samples	Obj. State Classes	Actions	Objects
Isola et al. [8]	Image	OS Classification	2015	N/A	63,440	9	–	18
OSDD [9]	Image	OS Classification & Detection	2021	N/A	19,000	9	–	18
Alayrac et al. [19]	Video	OS Classification & Action Localization	2017	Single	630	7	7	5
Fire et al. [65]	Video	SC Object Detection	2017	Single	490	17	–	13
Task-Fluent [18]	Video	OS Classification	2017	Single	809	21	14	25
ChangeIt [14]	Video	OSC Temporal Localization	2022	Single	34,428	–	44	–
HowToChange [35]	Video & Text	OSC Temporal Localization	2023	Single	498,475	20	–	134
VSCOS [61]	Video	SC Object Segmentation	2023	Single	1905	4	271	124
VOST [66]	Video	SC Object Segmentation	2023	Single	713	–	–	155
MOST [62]	Video	OS Classification	2024	Multiple	61	60	–	6
Ego4D [10]	Video	SC Object Detection & Classification	2022	Single	92,864	8	–	478
**Ego4D-OSCA**	Video	**OSC Anticipation**	**2026**	**Multiple**	**1498**	**9**	**5742**	**475**

**Table 4:** Per-class statistics for the Ego4D-OSCA dataset. The dataset contains a total of 63.923 training clips and 33.129 test clips.

	No OSC	Activate	Deactivate	Construct	Deconstruct	Deposit	Remove	Deform	Other
**Train**	2066	4017	1492	4186	1773	14,984	15,338	4400	15,667
**Test**	1284	1888	617	2289	966	7613	7608	2149	8715
**Percentage**	3.45%	6.09%	2.17%	6.67%	2.82%	23.28%	23.64%	6.75%	25.13%

**Table 5:** OSCA performance for various model configurations (O-: Oracle recognizer, VID-A: vision-only state anticipation model). Bold text indicates the best performance for the Oracle and the non-Oracle recognizer configurations.

Model	Top@1/5 mAcc	F1-Score
VID-A	23.93/89.10%	11.74%
VNLP (O-Action)	25.59/83.06%	24.62%
VNLP (O-State)	**40.07**/90.83%	33.57%
VNLP (O-Action, O-State)	39.20/89.76%	**37.12**%
VNLP (Action [71])	23.04/81.31%	22.09%
VNLP (State [9])	**32.72**/92.16%	21.78%
VNLP (Action, State)	29.42/94.65%	**26.29%**

**Table 7:** Robustness of the object state change anticipation model under varying recognition noise. Act.: Action, St.: State.

Noise (Act., St.)	Top@1/5 mAcc
(0%, 0%) (Oracle)	**39.20/89.76%**
(25%, 25%)	35.29/88.57%
(50%, 50%)	29.64/85.53%
(75%, 75%)	24.66/82.96%

**Table 8:** Asymmetrical history reduction impact.

Action	State	Acc. (Top@1/5)	F1-Score
50%	50%	**41.59%/90.95%**	36.85%
50%	25%	41.22%/91.10%	36.10%
25%	50%	41.23%/90.86%	**36.96%**
25%	25%	40.26%/90.79%	36.17%
100%	0%	25.59%/83.06%	24.62%
0%	100%	40.07%/90.83%	33.57%

**Table 9:** Top-1/Top-5 Accuracy and F1-score for evaluated Vision-Language Models (VLMs) against the proposed baseline. Bold text indicates the best performance per evaluation metric.

Model	Top@1/5 mAcc	F1-Score
Qwen2.5-VL-72B [75]	21.36%/82.01%	17.77%
Qwen2.5-VL-32B [75]	23.09%/79.49%	18.03%
InternVL2.5-38B [76]	22.54% / 80.67%	16.48%
Qwen2.5-VL-7B [75]	21.01%/63.10%	15.59%
InternVL3.5-8B [77]	20.78%/67.74%	17.55%
Qwen3-8B [78]	15.97%/71.50%	12.48%
LLaVA-NeXT-Video-34 [79]	18.49%/56.91%	11.73%
VNLP (Action, State) [Baseline]	29.42%/**94.65%**	26.29%
VNLP (O-Action, O-State) [Oracle]	**39.20%**/89.76%	**37.12%**

**Table 6:** Symmetrical history reduction impact. Bold text indicates the best performance per evaluation metric.

History Scale	Acc. (Top@1/5)	F1-Score
100% (1445)	39.20%/89.76%	**37.12%**
75% (1084)	39.74%/90.65%	36.08%
50% (722)	**41.59%/90.95%**	36.85%
25% (361)	40.26%/90.79%	36.17%
1% (14)	36.30%/88.21%	33.17%

**Figure A1:**
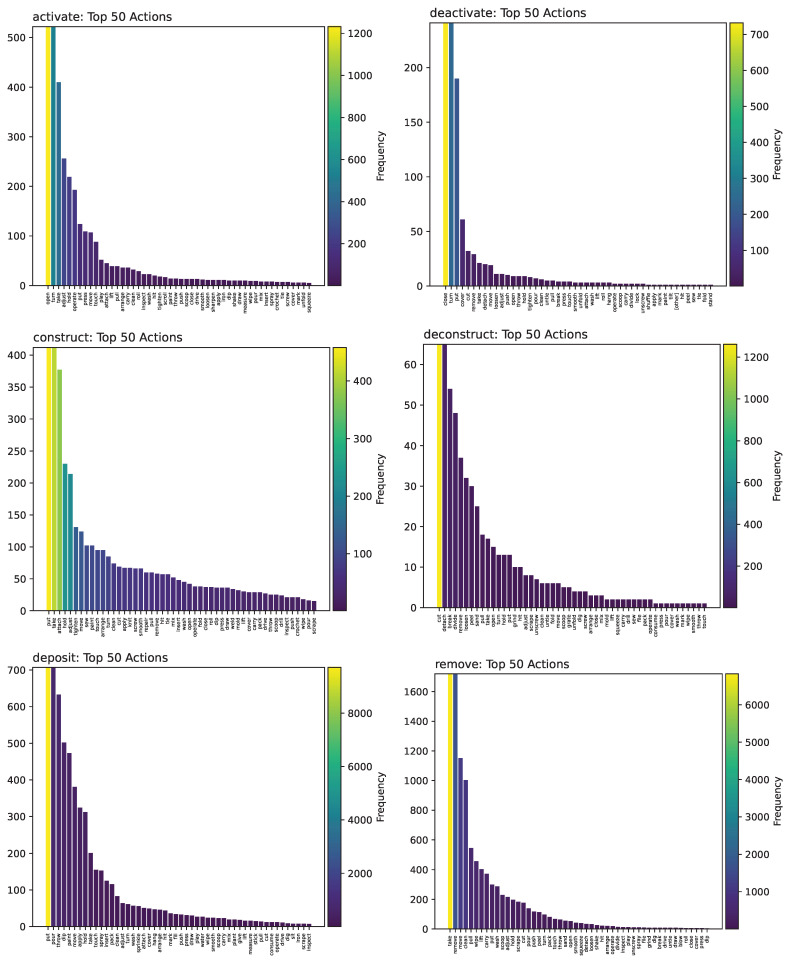
The frequency distribution of the top 50 actions (occurrences of action classes in the dataset action segments) concerning an object state change class is illustrated in each histogram for the classes ‘activate’, ‘deactivate’, ‘construct’, ‘deconstruct’, ‘deposit’, ‘remove’.

**Figure A3:**
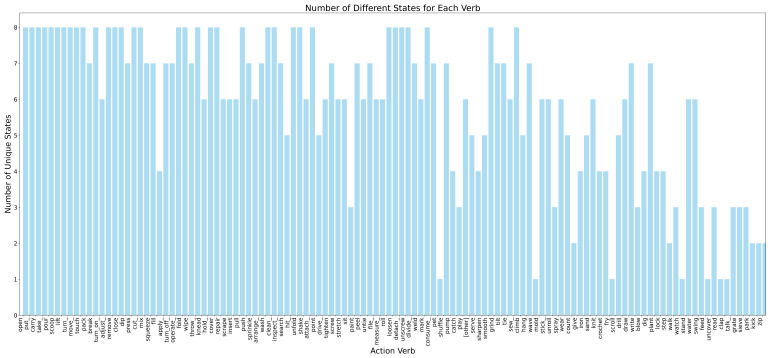
Histogram of object states associated with action verb classes.

**Figure A4:**
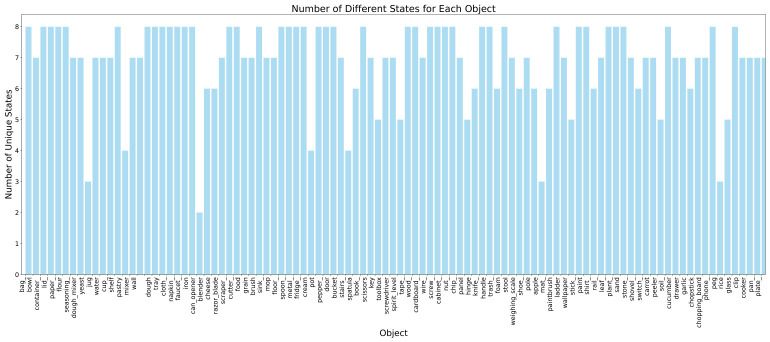
Histogram of object states associated with object classes. For better visualization purposes, we only depict the variability in the states of the first 100 objects.

**Figure A5:**
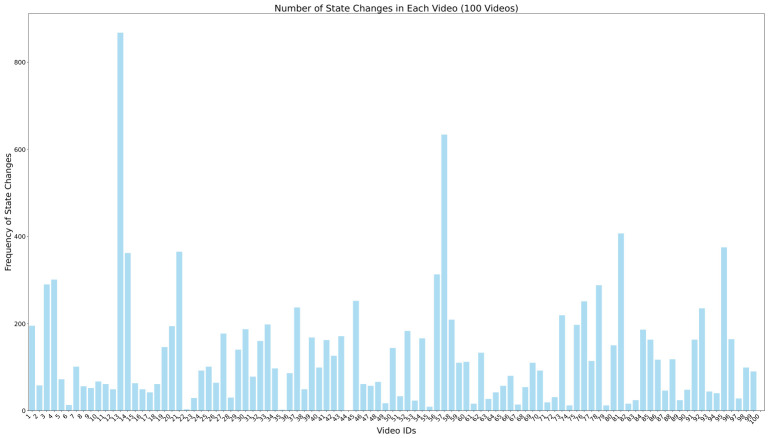
Histogram of the frequency of state transitions in the first 100 videos.

## Data Availability

The proposed dataset is freely available in https://github.com/FORTH-ICS-CVRL-HCCV/Ego4D-OSCA (last accessed on 1 July 2026).

## Appendix A

### Appendix A.1. Ego4D-OSCA Dataset Statistics

We focus on the dynamic nature of object interactions in the dataset by extracting statistics for the combinations of object states in conjunction with action verbs and object classes. We investigate how different action verbs are associated with a variety of object states, highlighting the diversity and context-dependence of an action’s effects. Additionally, we examine the variability of object states based on the actions performed, emphasizing the challenges imposed for the tasks of action and activity recognition and anticipation, and object state classification and anticipation. This analysis underscores the richness of the dataset and the sophisticated modeling required to accurately interpret and predict object states in various activity contexts.

#### Appendix A.1.1. Action Verbs vs. Object States

Each histogram illustrated in Figure A1 shows the distribution of the occurrences of action verbs in action segments of the proposed dataset associated with an object state change class, with challenging long-tail distributions. The high variability of action verbs and the state change class associations are observed for the ‘activate’, ‘deactivate’, ‘construct’, ‘deconstruct’, ‘deposit’, and ‘remove’.

Respectively, in Figure A2, each histogram shows the frequency of occurrences of the instances of object classes in action segments associated with an object state change class of the proposed dataset. In Figure A3, the histogram provides the number of distinct object state change classes associated with various action verb classes. It can be verified that the majority of action classes are associated with at least three distinct object state classes. For example, actions involving the verb “open” (leftmost label on the x-axis) can lead to any of the state change classes depending on the object, e.g., the “activate” state change occurs when opening a microwave and “deposit” when opening a box. The observed diversity highlights the complexity and context-dependence of state-modifying actions in the dataset, capturing a wide range of interactions and their state changes on interacting objects, which makes it valuable for training and testing sophisticated predictive models. This, in turn, indicates the need for elaborate models that can cope with the wide range of specific visual and semantic contexts in combination with different object classes involved in each action.

#### Appendix A.1.2. Objects vs. Object States

The histogram presented in Figure A4 illustrates that certain objects in *Ego4D-OSCA* can appear in up to eight different states, depending on the action performed, which reveals the action-dependent variability of object states within the dataset.

This observation underscores the complexity and dynamic nature of object interactions as well as the challenges to be tackled by solutions for classifying and predicting object state changes. The variability in these interactions presents significant challenges also for action recognition, where models need to accurately identify state changes and their transitions induced by subsequent actions, necessitating robust temporal models and comprehensive training data.

#### Appendix A.1.3. Statistics on State Changes Variability & Activity Duration

The histogram in Figure A5 illustrates the distribution of state transitions observed within the first 100 videos from the dataset. Each bar represents the frequency of specific state transitions between actions within a video, providing insights into the temporal dynamics and complexity of activities performed in the dataset.

The histogram highlights a significant variation in the number of state transitions observed within each video sample, indicating varying levels of complexity and duration in the actions performed. For instance, video 14 (video sample: *1e5bd816-e1dd-43d3-8709-42c83114dc7c*) stands out with 880 object state transitions and a duration of approximately 3 h. This underscores the intricate nature of the actions captured in the original Ego4D dataset [10], where the extent of state transitions, as presented in the proposed variant Ego4D-OSCA, reflects not only the complexity of the activities but also their temporal duration. Such variability emphasizes the need for comprehensive modeling approaches capable of accommodating diverse activity durations and complexities within the dataset. Moreover, it necessitates the consideration of a large action and state history by methods that utilize this information to predict future actions or object states, ensuring robustness and accuracy in forecasting.

Building on the significant diversity in the number of state transitions observed within each video, the transition matrix in Figure 9 provides further insight into the probabilistic nature of these transitions. For instance, the data indicate that when an object is in the ‘activate’ state during action *n*, it frequently transitions to the ‘deposit’ or ‘remove’ states in action n+1. This suggests that actions involving the activation of objects, typically electrical appliances, are usually followed by actions that involve placing items into or removing items from these objects. Such logical transitions underscore the presence of temporal action ordering and causality in activities, where one action sets the stage for subsequent actions. This pattern highlights the importance of understanding state persistence and transitions in developing predictive models. Accurately capturing these probabilistic dependencies is essential for models to effectively anticipate future states and actions. These insights reinforce the necessity for models to consider extensive action and state histories to accommodate the intricate and dynamic nature of the actions and activities in the Ego4D dataset, and inherently also in the proposed *Ego4D-OSCA*.

### Appendix A.2. Vision-Language Model Implementation Details

To ensure full reproducibility of our Vision-Language Model (VLM) baselines discussed in Section 5.4, we provide additional information on the implementation, frame sampling strategy, and exact prompt templates utilized during inference.

#### Inference Configuration and Sampling

All VLM inferences were conducted using an asynchronous client-server architecture powered by the SGLang framework to efficiently handle concurrent requests. For each video segment, we uniformly sampled up to N=16 frames from the available visual sequence to adhere to the context window limits of the evaluated models.

Generations were executed with a strict system instruction to output only the required reasoning and ranking blocks. The generation hyperparameters were held constant across all models to ensure a fair comparison:

- Maximum generated tokens: 512
- Temperature: 0.2
- Stop tokens: <|im_end|>, <|endoftext|>, </ranking>

### Appendix A.3. Vision-Language Model Characteristics and Inference Setup

To comprehensively evaluate the OSCA task and contextualize our baseline, we benchmarked a diverse set of state-of-the-art open-weights VLMs. All evaluated foundation models were sourced from the Hugging Face model hub. The selected models cover a wide spectrum of architectural paradigms and parameter scales, ranging from lightweight 8B models to 72B multi-modal reasoning architectures (including representatives from the Qwen, InternVL, and LLaVA model families).

To ensure high-throughput, reproducible, and isolated evaluation, model inference was executed on local multi-GPU clusters. We utilized the *vLLM* and *SGLang* inference engines to efficiently handle the heavy memory footprint of video-frame context windows. To fit hardware constraints while preserving evaluation integrity, large models were deployed utilizing tensor parallelism.

Table A1 summarizes the architectural characteristics, Hugging Face provenance, and specific inference deployment configurations for each evaluated model.

**Table A1:** Characteristics and inference configurations of the evaluated Vision-Language Models. All models were retrieved from the Hugging Face (HF) hub.

Model Family	Hugging Face Repository	Parameters	Max Context Window	Precision	Inference Engine
Qwen2.5-VL	Qwen/Qwen2.5-VL-72B-Instruct	72B	32,768	Default	SGLang
Qwen2.5-VL	Qwen/Qwen2.5-VL-32B-Instruct	32B	32,768	Default	SGLang
Qwen2.5-VL	Qwen/Qwen2.5-VL-7B-Instruct	7B	32,768	Default	SGLang
Qwen3-VL	Qwen/Qwen3-VL-8B-Instruct	8B	32,768	Default	SGLang
LLaVA-NeXT	llava-hf/LLaVA-NeXT-Video-34B-hf	34B	16,384	Default	vLLM
InternVL 2.5	OpenGVLab/InternVL2_5-38B	38B	32,768	Default	SGLang
InternVL 3.5	OpenGVLab/InternVL3_5-8B	8B	32,768	Default	SGLang

### Appendix A.4. Prompt Engineering

As the OSCA task requires reasoning over both visual evidence and historical context, we dynamically constructed the prompt for each video segment, as reported in Table A2. The prompt injected the chronologically ordered sequence of past actions and their corresponding object state changes directly into the text before the visual observation.

We utilized the zero-shot prompt template across all VLM evaluations. The model was constrained to rank the 9 possible candidate classes (including *no object state change*) to force strict adherence to the Ego4D-OSCA label space.

**Table A2:** The zero-shot prompt template utilized across all VLM evaluations for the OSCA task.

**System Prompt:** You are a procedural activity understanding expert specializing in sequential object state change prediction. You analyze short video clips of human actions within the context of an ongoing multi-step activity to forecast upcoming physical object state changes to objects. **TASK:** Given (1) a video clip of the most recently observed human action, (2) a complete chronological history of action labels and their corresponding object state changes, and (3) a fixed candidate label set—predict a ranked list of the object state change labels that will most likely occur during the NEXT, not-yet-observed action. **HARD CONSTRAINTS:** Your predictions MUST be labels copied verbatim from CANDIDATE LABELS. Predict the NEXT action’s state change only—NOT the current action. If you anticipate the next action will NOT produce a physical state change, rank “none” as the most likely. Rank all 9 candidates from most likely (1) to least likely (9). **<****action_history****>** OBSERVED ACTIONS (oldest → most recent): *{history_indexed}* **</****action_history****>** **<****object_state_change_history****>** CORRESPONDING OBJECT STATE CHANGES: *{history_bin_indexed}* **</****object_state_change_history****>** **<****candidate_labels****>** Activate, Deactivate, Deposit, Remove, Construct, Deconstruct, Deform, Other, None **</****candidate_labels****>** **Reason through the following before predicting:** 1. What complex activity is being performed, and what is its overall goal? 2. What is the cumulative object state after all observed actions? 3. What specific action is shown in the video, and what contextual cues does it provide? 4. What action would most plausibly follow next? 5. What object state changes would that next action produce? 6. Rank the candidate labels based on how closely they match. **OBSERVATION:** (The following images are sampled frames from the most recently observed action) *[Image Tokens Inserted Here]*
